# Recent Treatment Strategies for Acute Pancreatitis

**DOI:** 10.3390/jcm13040978

**Published:** 2024-02-08

**Authors:** Yongcook Song, Sang-Hoon Lee

**Affiliations:** Department of Internal Medicine, Konkuk University School of Medicine, Seoul 05030, Republic of Korea; 20230178@kuh.ac.kr

**Keywords:** acute pancreatitis, fluid resuscitation, initial management

## Abstract

Acute pancreatitis (AP) is a leading gastrointestinal disease that causes hospitalization. Initial management in the first 72 h after the diagnosis of AP is pivotal, which can influence the clinical outcomes of the disease. Initial management, including assessment of disease severity, fluid resuscitation, pain control, nutritional support, antibiotic use, and endoscopic retrograde cholangiopancreatography (ERCP) in gallstone pancreatitis, plays a fundamental role in AP treatment. Recent updates for fluid resuscitation, including treatment goals, the type, rate, volume, and duration, have triggered a paradigm shift from aggressive hydration with normal saline to goal-directed and non-aggressive hydration with lactated Ringer’s solution. Evidence of the clinical benefit of early enteral feeding is becoming definitive. The routine use of prophylactic antibiotics is generally limited, and the procalcitonin-based algorithm of antibiotic use has recently been investigated to distinguish between inflammation and infection in patients with AP. Although urgent ERCP (within 24 h) should be performed for patients with gallstone pancreatitis and cholangitis, urgent ERCP is not indicated in patients without cholangitis. The management approach for patients with local complications of AP, particularly those with infected necrotizing pancreatitis, is discussed in detail, including indications, timing, anatomical considerations, and selection of intervention methods. Furthermore, convalescent treatment, including cholecystectomy in gallstone pancreatitis, lipid-lowering medications in hypertriglyceridemia-induced AP, and alcohol intervention in alcoholic pancreatitis, is also important for improving the prognosis and preventing recurrence in patients with AP. This review focuses on recent updates on the initial and convalescent management strategies for AP.

## 1. Introduction

Acute pancreatitis (AP) is an acute inflammation of the pancreas, a vital organ responsible for the production of digestive enzymes and hormones that regulate blood sugar levels. This disease is characterized by the premature activation of digestive enzymes within the pancreas, leading to autodigestion and inflammation. AP is a leading gastrointestinal disease that causes hospitalization worldwide, and its incidence is increasing in many countries [1,2]. Among patients who are admitted with AP, around 80% have a mild clinical course; however, others develop severe disease, with a mortality rate of approximately 20% [2].

Initial management in the first 72 h after diagnosis is pivotal and can influence the clinical course and outcome of the disease. Early identification and appropriate intervention can prevent complications and improve patient outcomes. Although there is no specific pharmacological therapy available for AP, initial management, including assessment of disease severity, fluid resuscitation, pain control, nutritional support, antibiotic use, and endoscopic retrograde cholangiopancreatography (ERCP) in gallstone pancreatitis, plays a fundamental role in AP treatment. Furthermore, convalescent treatment is important for improving prognosis and preventing recurrence in patients with AP [3]. This review focuses on recent updates on the initial and convalescent management strategies for AP.

## 2. Initial Management during the First 72 h

### 2.1. Assessment of Disease Severity

After the initial diagnosis of AP, it is crucial to assess the severity of AP in order to predict the likelihood of a severe clinical course, which may involve organ failure and even mortality. In addition, this assessment is necessary to determine appropriate initial management and treatment strategies for the future.

The severity of AP is determined by the development of organ failure(s) and local complications, which are mostly classified according to the Revised Atlanta Classification [4]. Severe AP, defined as persistent organ failure (lasting > 48 h), can result in a mortality rate of up to 43% during the initial attack [5]. Patients with severe AP require intensive care unit monitoring and support for circulatory, pulmonary, renal, and hepatobiliary function to reduce the risk of organ failure sequelae.

Many prognostic models, including patient-related risk factors, laboratory parameters and scoring systems, have been developed to predict severe AP early in the disease course [6,7,8,9,10,11,12,13,14]. Although numerous predictive tools are available, no approach has emerged as definitively superior to others in large-scale comparisons [13,15,16]. Unfortunately, our ability to predict the severity of AP early remains limited (accuracy of approximately 80%) [15,17]. Among all prognostic tools, systemic inflammatory response syndrome (SIRS) is a commonly used, validated predictor, which may be as accurate as other more complicated scores, and the absence of SIRS on day 1 is associated with a high negative predictive value [18,19]. Another easily applicable score is the bedside index of severity of acute pancreatitis (BISAP) score. A BISAP score of ≥3 was significantly associated with an increased risk of mortality [14]. Recently, an artificial intelligence model named EASY-APP was developed as a web-based application that can easily identify patients at high risk for severe AP within hours of hospitalization [20]. Especially during the initial phase of AP, these predictive parameters should be followed serially to monitor the clinical course and treatment response. 

### 2.2. Fluid Resuscitation

Traditionally, intravenous fluid resuscitation stands out as a cornerstone in AP management of any severity [17]. In patients with AP, increased vascular permeability and decreased osmotic pressure cause extracellular fluid leakages around the pancreas and into the retroperitoneal, abdominal, and thoracic cavities, resulting in a significant loss of circulating plasma volume. This can lead to hypovolemia and hypoperfusion, even resulting in other organ failures in severe AP. Therefore, early and adequate fluid resuscitation is important to stabilize cardiovascular distress and increase pancreatic microcirculation. Several previous studies have demonstrated that initial aggressive fluid resuscitation can improve survival by minimizing pancreatic necrosis [21,22,23,24]. However, over-aggressive fluid therapy can be associated with poor clinical outcomes in patients with severe AP, including sepsis, respiratory complications, and abdominal compartment syndrome [25,26]. At present, there are no clearly defined details regarding the goal of fluid resuscitation, fluid type, rate, volume, and duration [27].

#### 2.2.1. Goal-Directed Therapy (GDT)

In patients with AP, several current guidelines suggest using GDT for fluid resuscitation [28,29,30]. GDT refers to the titration of intravenous fluids to specific clinical and biochemical targets of perfusion, such as the heart rate (HR), mean arterial pressure (MAP), central venous pressure (CVP), urine output (UO), central venous oxygen saturation (ScvO2), blood urea nitrogen (BUN) concentration, hematocrit, and lactate levels [28]. Although GDT did not result in significantly improved mortality or a decrease in the rate of persistent multiple organ failure [27], it has been considered to be a structured approach in which fluid administration is guided by specific physiological targets rather than empirical estimates, especially in patients with severe AP. These “goals” are determined by using various hemodynamic and biochemical parameters that reflect the patient’s volume status and perfusion (Table 1).

In patients with severe AP with organ failures requiring ICU admission, a more tailored and individualized approach to fluid resuscitation is required to avoid under- or over-treatment. Because a single clinical parameter alone is unlikely to reflect the overall volume status, simultaneous assessment of multiple parameters according to each phase of early AP is more reasonable [34]. These patients should be frequently assessed, ideally every 2–3 h, to adjust fluid therapy based on changes in these parameters. A recent pilot study showed that fluid therapy protocols based on dynamic parameters and tests (stroke volume changes after mini-fluid challenge (250 mL normal saline within 10 min) and passive leg raising test) were more reliable in predicting fluid responsiveness in patients with predicted severe AP [35].

#### 2.2.2. Fluid Type

The fluid type for resuscitation in AP is an isotonic crystalloid solution, which contains normal saline (NS) and balanced/buffered crystalloids (such as lactated Ringer’s (RL), Plasma-Lyte, or Hartmann’s solution). Although NS has traditionally been used for fluid resuscitation in AP, concerns have been raised regarding the adverse effects of NS, such as hyperchloremic non-anion gap acidosis and acute kidney injury. Regarding clinical evidence of fluid resuscitation using RL vs. NS, a meta-analysis demonstrated that the LR group was less likely than the NS group to progress to moderately severe or severe AP, requiring ICU admission or developing local complications [36]. The results of two large RCTs published in 2018 suggested that balanced crystalloids (LR or Plasma-Lyte) were favored over NS. The SMART study found that the use of balanced crystalloids can reduce the composite outcomes of in-hospital mortality, new renal replacement therapy, and persistent renal dysfunction in critically ill patients [37]. Another SALT-ED trial of non-critically ill patients in the emergency department revealed that balanced crystalloids resulted in a significant decrease in major adverse kidney events within 30 days, without a difference in hospital-free days [38]. Additionally, the use of LR could be associated with an anti-inflammatory effect, as shown by the decrease in C-reactive protein levels and incidence of SIRS [39]. Although the panel disagreed with the superiority of RL over NS in the AGA guidelines due to the low quality of evidence for major clinical outcomes [28], the clinical benefits of using RL are believed to outweigh the risks. Further detailed prospective comparative studies are warranted.

The use of colloids, including ‘semi-synthetic’ colloids (hydroxyethyl starch (HES), gelatin and dextran solutions) and ‘natural’ colloids (human albumin solution), is not recommended because of potential adverse effects without a demonstrable survival benefit [22,40,41]. The CHEST trial, a blinded, randomized, controlled trial comparing crystalloid and HES, showed that acute kidney injury and adverse events (pruritis and skin rash) were more common in the HES group than in the NS group [40]. In addition, intravenous albumin infusion did not improve the clinical prognosis of patients with AP [42].

#### 2.2.3. Fluid Rate and Volume

Early aggressive hydration has been widely recommended for the initial management of AP [28,29,30,43]. However, there are controversies regarding optimal fluid volume and infusion rate. Several RCTs subsequently compared aggressive and non-aggressive fluid resuscitation (Table 2). The results from the first two RCTs conducted in China for patients with severe AP demonstrated worse clinical outcomes with aggressive fluid therapy [44,45]. Wu et al. did not observe any differences between GDT and standard fluid therapy [46]. Although Buxbaum et al. demonstrated that aggressive fluid hydration appeared to be effective in mild AP [24], a recent large RCT of 249 patients with mild AP (WATERFALL study) was terminated early because of safety issues regarding whether aggressive fluid resuscitation was associated with an increased incidence of fluid overload (20.5% vs. 6.3%) without improvement in clinical outcomes [23]. 

Based on the available evidence from RCT results and several guidelines, we recommend a moderate fluid resuscitation strategy, starting with 1.5 mL/kg/h of LR infusion rate, preceded by bolus of 10–20 mL/kg over 2 h if patients have moderately severe to severe AP, signs of hypovolemia, acute kidney injury, or poor predictive indicators, such as hematocrit ≥ 44% or BUN > 25 mg/dL. The following fluid volumes are generally considered appropriate for the initial management of AP: 3 L at 24 h and 4–6 L at 48 h for mild AP; 3–4 L at 24 h and 6–8 L at 48 h based on clinical/laboratory parameters for moderate or severe AP [48].

#### 2.2.4. Fluid Therapy Duration

In most patients with mild AP, oral feeding can be initiated 12 h after AP onset if abdominal pain is low, and fluid resuscitation can be stopped once the patient tolerates oral feeding. When patients are suspected to experience volume overload, the fluid should be decreased or stopped. The duration of fluid therapy might be longer in moderate to severe AP patients and is guided by the patient’s clinical status, including factors such as hemodynamic stability, organ function, and resolution of symptoms. Continuous monitoring with GDT is essential for adjusting fluid therapy as needed.

### 2.3. Pain Control

The primary symptom of AP is abdominal pain, often severe and persistent, which requires effective management. Pain control is a pivotal element in the multidisciplinary management of AP; however, no single analgesic strategy has been universally accepted as superior in terms of efficacy and safety.

#### 2.3.1. Opioids

Historically, there has been hesitation to use opioids for AP patients due to concerns about inducing sphincter of Oddi spasm. However, recent evidence suggests that opioids can be safely used in AP without increasing the risk of adverse events related to the sphincter of Oddi [49]. Opioids provide potent analgesia and are particularly effective in managing severe pain associated with AP. Their rapid onset of action and efficacy in reducing visceral pain make them a preferred choice in many clinical scenarios. Although opioids are effective, they are associated with a risk of respiratory depression, constipation, and potential for dependence. However, in the context of AP, short-term use is generally considered safe [50]. Recently, a comparative RCT evaluated the efficacy and safety of intravenous buprenorphine (a more potent opioid than morphine with less respiratory depression and abuse potential) vs. IV diclofenac (a non-steroidal anti-inflammatory drugs (NSAIDs)) for analgesia in AP patients demonstrated that buprenorphine appears to be more effective and equally safe, even in the subgroup of patients with moderately severe or severe pancreatitis [51].

#### 2.3.2. NSAIDs and Acetaminophen

NSAIDs and acetaminophen, especially intravenous formulations, such as dexketoprofen, diclofenac and paracetamol, offer an alternative to opioids. They can be particularly useful in patients whom opioid use might be contraindicated or in those at risk of opioid-related side effects. Studies have indicated that NSAIDs, particularly paracetamol, can provide analgesia comparable to that of opioids in AP. Their anti-inflammatory properties may offer additional benefits in the context of pancreatitis [49]. NSAIDs and acetaminophen are generally well-tolerated. However, they should be used with caution in patients with renal impairment, gastric ulcers, or those at risk of bleeding. 

#### 2.3.3. Epidural Analgesia

Epidural analgesia, particularly thoracic epidural analgesia, has been explored for pain management in patients with AP admitted to the ICU. It has been associated with decreased mortality in a multicenter retrospective propensity analysis. In an EPIPAN multicenter RCT, thoracic epidural analgesia was investigated in ICU patients with AP. The trial suggested potential benefits, including improved pancreatic perfusion and decreased AP severity, with no significant difference in adverse events attributable to epidural analgesia in ICU patients with severe AP [52].

### 2.4. Nutritional Support

Traditionally, the “pancreatic rest” concept has been suggested as the initial management of AP to avoid pain and pancreatitis aggravation. However, recent research has also shown that early oral or enteral feeding results in shorter hospital stays, fewer complications, and lower mortality rates in patients with AP [53,54,55]. One study comparing parenteral and enteral nutrition revealed that oral feeding can reduce sepsis and AP severity. These clinical benefits may result from preventing atrophy of the gastrointestinal mucosa and maintaining the function of the gut-mucosal barrier, thereby reducing bacterial translocation and minimizing the risk of infected peripancreatic necrosis [56]. 

#### 2.4.1. When to Start Oral Feeding

A pooled analysis of the results of 11 RCTs that addressed the role of early vs. delayed feeding demonstrated that when started within 48 h of admission, enteral nutrition resulted in a significant reduction in the risks of multiple organ failure, pancreatic infectious complications, and mortality, compared with parenteral nutrition [55]. Therefore, most guidelines recommend early (within 24–48 h) oral feeding rather than keeping the patient nil per os (NPO), especially if patients are pain-free and their laboratory parameters have improved [28,29,30,43]. 

The PYTHON trial, a multicenter, randomized, controlled superiority trial, aimed to compare the outcomes of early naso-enteric tube feeding (within 24 h of randomization; early group) to an oral diet that starts at 72 h after presentation (tube feeding provided if the oral diet was not tolerated; on-demand group) in patients diagnosed with AP. This study did not show a significant difference in clinical outcomes (major infection or death) between the early and on-demand groups [57]. The recent PADI trial focused on determining the optimal time to start oral refeeding in patients with mild and moderate AP to reduce hospital length of stay (LOS) and its complications. This trial compared immediate oral refeeding (low-fat-solid diet initiated immediately after hospital admission) with conventional oral refeeding (fasting for the first 24 to 48 h and resuming oral diet when clinical and laboratory parameters improved), highlighting the benefits of immediate oral refeeding in reducing hospital stay and cost savings with fewer complications. The authors, therefore, asserted to start an oral diet without waiting for improvement of clinical symptoms and laboratory findings in patients with mild or moderate AP [58].

#### 2.4.2. Route of Tube Feeding

The current meta-analysis and guidelines strongly favor enteral over parenteral nutrition [28,55]. However, some patients who are intolerant of oral feeding within 72 h due to pain, vomiting and ileus may require the placement of an enteral tube for nutritional support. The studies, including three RCTs, which specifically addressed the issue of nasogastric vs. nasoenteral (either nasoduodenal or nasojejunal) feeding, did not demonstrate a clinical benefit related to the route for tube feeding, either in mild or severe AP [59]. A nasogastric tube is relatively easy to insert compared to a nasoenteral tube. Both feeding routes can be selected depending on the patient’s condition. Parenteral nutrition is indicated only when the enteral route is impossible or unable to meet the minimum calorie requirements.

### 2.5. Prophylactic Antibiotic Use

The pathophysiology of necrotizing pancreatitis is marked by pancreatic necrosis, which is vulnerable to microbial colonization of non-viable pancreatic tissue, resulting in infected necrosis. Infected necrosis is highly associated with mortality in the late phase of AP (approximately 30%), and mortality doubles when infected necrosis coexists with organ failure [43,60]. To mitigate the risk of infected necrosis, morbidity, and mortality in patients with predicted severe AP or diagnosed with necrotizing pancreatitis, a series of RCTs evaluated prophylactic antibiotic use before documented infection. While earlier trials and meta-analyses often showed improvement in clinical outcomes by prophylactic antibiotic use, more recent studies and subsequent meta-analyses consistently failed to demonstrate consistent evidence of benefit from antibiotic prophylaxis [61,62]. Consequently, the use of prophylactic antibiotics to reduce the frequency of infection-related complications or mortality in AP, including severe and necrotizing pancreatitis, remains underpowered, and further large randomized controlled trials are warranted.

#### Procalcitonin-Guided Antibiotic Use

**Figure 1:**
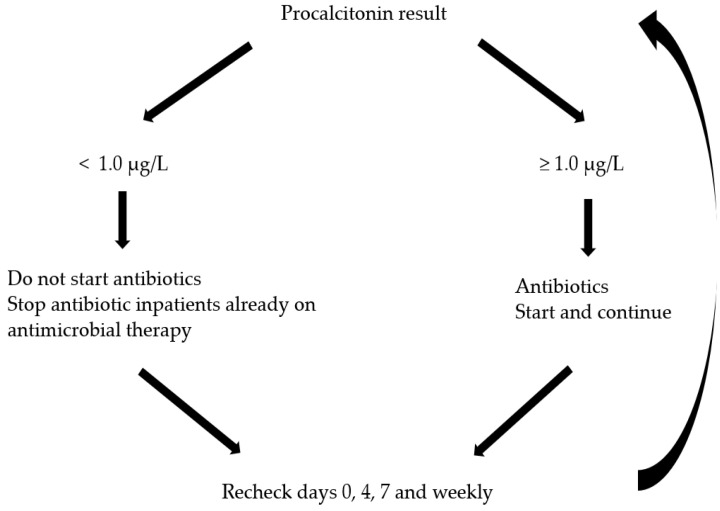
Procalcitonin-based algorithm in RPOCAP trail [64].

The decision-making process regarding antibiotic use is challenging, especially in the setting of an AP patient presenting with systemic symptoms such as fever, leukocytosis, and elevated C-reactive protein levels. None of these features distinguish between inflammation and infection, leading to global overuse of antibiotics during AP hospitalization [62,63]. The PROCAP trial, the largest randomized trial to date, investigated the use of a procalcitonin algorithm (Figure 1) to guide antibiotic use in patients with AP. The study showed that procalcitonin-guided care significantly decreased the probability of being prescribed an antibiotic without increasing the risk of infection or harm to AP patients [64].

### 2.6. Timing and Role of ERCP and Endoscopic Ultrasonography (EUS) in Gallstone Pancreatitis

Gallstones are the most common cause of AP, which is clinically initiated by the impaction of gallstone stones or sludges in the common bile duct or ampulla [65,66]. Patients with gallstone pancreatitis may develop cholangitis, organ failure, and other life-threatening complications. ERCP quickly addresses the gallstone and provides rapid biliary decompression, thereby alleviating the severity of pancreatitis. Urgent ERCP (within 24 h of admission) should be performed in patients with gallstone pancreatitis and concomitant cholangitis [67,68]. For patients with gallstone pancreatitis without cholangitis, the optimal timing for therapeutic ERCP may be 24–48 h after their diagnosis (24 h to allow spontaneous passage of stones and 48 h to avoid prolonged biliary obstruction) [59]. When in doubt about biliary obstruction without cholangitis, magnetic resonance cholangiopancreatography (MRCP) or EUS could be performed to determine the presence of common bile duct stones and the necessity for ERCP to remove them. 

In a systemic review of eight RCTs, early ERCP in patients without cholangitis did not reduce the risk of overall pancreatic complications, organ failure or death [59]. Especially among patients with predicted severe acute biliary pancreatitis but without cholangitis, the results of recent APEC and APEC-2 trials showed that urgent ERCP, even when guided by EUS to select patients with biliary stones and sludges in the APEC-2 trial, failed to significantly reduce major complications or mortality compared to conservative treatment [69,70]. As a result, there has been a growing inclination towards a more conservative strategy, reserving ERCP for cases where there is a clear indication, such as the presence of cholangitis or persistent biliary obstruction. This shift reflects a broader trend in practice towards more personalized care, where treatments are increasingly tailored to the specific needs and circumstances of individual patients with gallstone pancreatitis.

### 2.7. Other Therapeutic Interventions

#### 2.7.1. Insulin and Plasmapheresis for Hypertriglyceridemia Induced AP (HTG-AP) 

HTG-AP occurs when excessively high levels of triglycerides (TGs) in the blood lead to increased blood viscosity, capillary blockage in the pancreas, and the release of toxic free fatty acids. Insulin lowers TG levels by enhancing lipoprotein lipase activity, and plasmapheresis can rapidly remove TGs and free fatty acids from the blood. No RCTs have addressed their efficacy and safety. A few recent meta-analyses, largely based on observational studies, indicate that these treatments are effective in accelerating TG level reduction (<500 mg/dL) but do not affect mortality compared with conventional management [71,72,73,74]. The ELEFANT trial is an ongoing randomized controlled trial that investigates the concept that the early elimination of TG and free fatty acids from the blood is beneficial in HTG-AP [75]. This study will provide evidence for early lipid-lowering interventions in HTG-AP management.

#### 2.7.2. Low-Molecular-Weight Heparin (LMWH)

LMWHs, such as enoxaparin, are anticoagulants that prevent the formation of blood clots. Their role in AP is based on the premise that microvascular thrombosis plays a role in the disease progression. A recent randomized, single-bind, phase 3 control trial emphasized the potential benefits of LMWH in AP. The study found that LMWH can reduce necrosis of the pancreas, especially in the early phase of moderate and severe AP [76].

#### 2.7.3. Protease Inhibitors

Protease inhibitors are used to treat AP; their effectiveness is a topic of debate. These inhibitors prevent the activation of enzymes that can damage the pancreas. A meta-analysis aimed to determine the effectiveness of protease inhibitors in reducing mortality or morbidity associated with AP. Overall, treatment with protease inhibitors did not significantly reduce the mortality rate associated with AP [77,78].

## 3. Management of Local Complications

Local complications of AP include acute peripancreatic fluid collection, pancreatic pseudocysts, acute necrotic collection, and walled-off necrosis (WON). The development of acute peripancreatic fluid collections and acute necrotic collections typically occurs within the first four weeks of AP, whereas the formation of pancreatic pseudocysts and WON usually occurs with encapsulation >4 weeks after AP onset [4]. Although the revised Atlanta classification indicates that WON typically develops >4 weeks, over 40% of demarcated necrotic collections had already developed within the first 3 weeks after the onset of necrotizing pancreatitis [79]. 

In actual clinical practice, local complications should be suspected when there is persistent or recurring abdominal pain, secondary increases in serum pancreatic enzyme activity, development of clinical signs of sepsis, such as fever and leukocytosis, worsening organ dysfunction, and/or clinical failure to improve after 7–10 days of hospitalization. In such cases, prompt contrast-enhanced abdominal CT and/or MRI should be performed to confirm the diagnosis of local complications and infection. 

### 3.1. Local Complications in Interstitial Edematous Pancreatitis

In a longitudinal study of interstitial pancreatitis, most acute peripancreatic fluid collections spontaneously resolved within 7–10 days, and only 6.8% lasted beyond four weeks, resulting in the development of pancreatic pseudocysts [80]. In addition, the spontaneous resolution of pancreatic pseudocysts is common, with reported rates of up to 70% [81].

#### 3.1.1. Indication of Intervention for Pancreatic Pseudocysts

The drainage of mature pseudocysts is indicated in patients with symptoms (persistent abdominal pain, nausea, early satiety, anorexia, weight loss, or jaundice) or complications (infection, bleeding, or obstruction (gastric, duodenal, or biliary obstruction)), regardless of pseudocyst size.

#### 3.1.2. Method of Intervention for Pancreatic Pseudocysts

EUS-guided transmural drainage is more commonly performed than surgery or percutaneous drainage in patients with symptomatic or complicated pancreatic pseudocysts abutting the stomach or duodenum. This is because transmural drainage has been shown to be effective in resolving pseudocysts, with a lower incidence than surgery and without the need for external drains [82,83,84]. For selected patients (e.g., those with a pseudocyst communicating with the main pancreatic duct or those with pancreatic duct stricture), ERCP-guided placement of a transpapillary pancreatic stent can be performed [85]. 

### 3.2. Necrotizing Pancreatitis

Pancreatic necrosis is defined as non-enhancement of the pancreatic parenchyma on contrast-enhanced CT, and necrotizing pancreatitis manifests as necrosis involving the pancreas alone, extra-pancreatic tissue alone, or most commonly, both [4,43,86]. It is important to note that contrast-enhanced CT within 48–72 h after the onset of AP cannot exclude the presence of pancreatic necrosis. Therefore, if necrotizing pancreatitis is suspected, it should be assessed at least three days after presentation. Accurate classification of local fluid collections is important because the management and prognosis of necrotizing pancreatitis are significantly more challenging and unfavorable than those of intestinal edematous pancreatitis.

#### 3.2.1. Infected Necrosis

Infected necrosis occurs as a complication in approximately one-third of patients with necrotizing pancreatitis, most commonly 2–4 weeks after AP presentation [87]. Both acute necrotic collection and WON are initially sterile but can become infected over time. This is thought to result from the bacterial translocation from the gut to the adjacent necrotic pancreatic parenchyma. 

Infected necrosis has a high mortality rate of 30% and is a leading cause of morbidity and mortality in necrotizing pancreatitis [86]. Therefore, when infection is strongly suspected (e.g., gas in necrosis, bacteremia, sepsis, or clinical deterioration), empiric antibiotic therapy is promptly initiated without culture or aspiration [43,86]. Broad-spectrum intravenous antibiotics known to penetrate pancreatic necrosis (for example, a carbapenem alone or a quinolone, ceftazidime, or cefepime combined with anaerobic coverage, such as metronidazole) should be favored. Further therapeutic interventions can be explored, and the appropriate strategies, indications, timing, and methods for such interventions are discussed below.

- Diagnosis of infected necrosis

Abdominal computed tomography (CT) images showing the presence of an extraluminal gas configuration within the area of necrosis were regarded as pathognomonic. However, it is only found in approximately half of patients with infected necrosis, and the absence of gas does not signify the absence of infection [79,88,89]. 

EUS- or CT-guided fine-needle aspiration (FNA) of the necrotic collection for Gram staining and culture can be performed to confirm the presence of infection. However, this diagnostic procedure is unnecessary in the majority of cases, and recent guidelines do not recommend the routine use of FNA [29,43,90] for the following reasons. First, in a prospective, multicenter database of 208 consecutive patients, a post hoc analysis revealed that 80–94% of infected necrosis cases were diagnosed based on clinical or imaging studies without FNA results, and their mortality was not different between the groups [88]. Second, the diagnosis of infected necrosis through early FNA is not necessary for clinical decision-making regarding interventions. In current practice, therapeutic interventions are postponed whenever clinically feasible until necrosis becomes encapsulated [90,91,92]. In addition, false-negative results are possible in approximately 25% of cases, and there is a theoretical risk of contaminating a sterile collection exit [88,89,93]. For patients with (peri-)pancreatic collections who exhibit clinical deterioration or fever in the absence of any other infection focus, such as pulmonary, urinary tract, or line infections, a presumptive diagnosis of infected necrosis is justifiable.

#### 3.2.2. Treatment Strategies for Necrotizing Pancreatitis

Advances in our understanding of the pathophysiology and natural course of necrotizing pancreatitis, along with developments in therapeutic intervention techniques, have led to a significant paradigm shift in the treatment strategies for the disease. In the 1980s, necrotizing pancreatitis was mainly treated by surgeons performing necrosectomy within 1–3 days of onset [94]. However, the results of the PANTER trial, presented in 2010, demonstrated that a minimally invasive ‘step-up’ approach is better than an open necrosectomy with a significant decrease in the rate of new-onset multiple organ failure (12% vs. 40%), incisional hernia (6% vs. 19%), and new-onset diabetes (16% vs. 38%) [95]. The step-up approach in the PANTER trial consisted of percutaneous drainage followed, if needed, via minimally invasive retroperitoneal necrosectomy (usually after 4 weeks). Interestingly, in the step-up approach group, 35% of patients were successfully managed with percutaneous drainage only. The traditional management of infected necrosis with upfront surgical debridement has been almost completely replaced by minimally invasive surgical and endoscopic step-up approaches.

Recent treatment strategies for necrotizing pancreatitis conceptually consist of four steps: (1) conservative treatment with antibiotics; (2) percutaneous or endoscopic transmural drainage; (3) minimally invasive necrosectomy, either video-assisted retroperitoneal debridement (VARD) or endoscopic necrosectomy; and (4) open necrosectomy. Detailed indications, timing, anatomical considerations, and selection of each intervention method are discussed below.

- Indications of intervention

Pancreatic necrosis can lead to secondary infection or symptomatic sterile necrosis, which includes intestinal or biliary obstruction, worsening organ failure, and persistent unwellness of the patient. Both infected necrosis and symptomatic sterile necrosis are accepted indications for therapeutic interventions. If the signs of infection continue despite receiving antibiotics for 48 to 72 h, it is necessary to consider interventional techniques for draining the collection as the next step. Asymptomatic patients with sterile pancreatic necrosis are usually observed, as the risk of iatrogenic complications during the procedure is much higher than that of spontaneous complications arising from fluid collection [96].

- Timing of intervention

Pancreatic intervention should be optimally delayed for 4 weeks until pancreatic necrosis has become encapsulated. During the first few weeks of the AP phase (<3 to 4 weeks), we attempt to postpone the procedure by continuing antibiotics for at least 4 weeks and reserving catheter drainage in patients who are experiencing clinically ongoing deterioration. The results of the POINTER trial revealed that routine immediate drainage, even when infected necrosis was diagnosed within the first 4 weeks, did not improve clinical outcomes but actually led to more invasive interventions (catheter drainage and necrosectomy) compared with the postponed drainage group. In fact, 39% of infected necrosis cases only improved with antibiotics without the need for any other intervention, suggesting that initial conservative management with antibiotics and a postponed drainage strategy are justified when infected necrosis is diagnosed, and help prevent unnecessary procedures, especially in the early phase of AP [92].

- Anatomical considerations for intervention

The location of pancreatic necrosis, as assessed via preprocedural cross-sectional imaging, is a key factor in guiding approaches to pancreatic intervention [90]. Central collections located within the lesser sac abutting the posterior gastric wall can be assessed through the transgastric route either endoscopically, laparoscopically, or open. Endoscopic debridement is generally preferred because it is associated with fewer complications than surgical approaches. Retrogastric collections that extend deep into the left paracolic gutter can be drained via a left retroperitoneal approach, initiating with percutaneous drainage followed by VRAD or minimally invasive retroperitoneal pancreatic debridement (MIRP), if necessary. Alternatively, these collections can be drained endoscopically, and additional percutaneous drains can be used to address the dependent component in the left paracolic gutter. Collections located in the root of the mesentery or to the right of the mesenteric vessels are challenging to access through percutaneous, endoscopic, or retroperitoneal approaches. In such cases, laparoscopic transperitoneal or traditional open approaches may be necessary [97].

#### 3.2.3. Selection of Interventional Techniques

The decision to perform drainage and/or necrosectomy in patients with necrotizing pancreatitis is individualized and takes into account various factors, such as the patient’s status (hemodynamic stability, symptoms, laboratory findings, comorbidities, and clinical course), characteristics of necrosis (the presence of a mature encapsulated wall, the amount of necrotic debris, location, extent, and distance from the gastrointestinal tract), and procedural factors (the advantages and disadvantages of each intervention, including endoscopic, percutaneous, or surgical, and their combinations as the step-up approach).

- Percutaneous drainage

Temporary percutaneous catheter drainage can be used as a primary modality, as an initial procedure in the step-up approach, or as a bridging therapy even in the early phase of AP before 4 weeks when surgical debridement is highly morbid. After 4 weeks, endoscopic drainage is preferred as a much lower rate of pancreatic fistula than percutaneous drainage [83,98]. Percutaneous drainage is usually reserved for salvage management when endoscopic drainage is unsuccessful or not technically feasible.

In general, the retroperitoneal route is preferred because it avoids enteric leaks and peritoneal contamination and can be used later for VARD, MIRP, or percutaneous endoscopic necrosectomy. After the placement of single or multiple catheters, the catheter underwent vigorous manual irrigation with isotonic saline and was serially upsized to larger-bore catheters and repositioned to easily remove necrotic debris.

A systemic review of 11 studies with 384 patients revealed an overall success rate of 56% when percutaneous drainage was used as the primary drainage for necrotizing pancreatitis. Adverse events such as external fistulae occurred in up to 27% of the patients [99].

- Endoscopic drainage

Endoscopic transmural drainage involves the creation of a fistula into necrotic cavities using EUS rather than direct puncture under endoscopic vision. EUS was associated with higher technical success (95% vs. 35–66%) and a trend toward lower adverse event rates (0–4% vs. 13–15%) than the conventional direct puncture technique in two randomized controlled trials [100,101]. EUS enables the visualization and puncture of targeted collections independent of a visible bulge, and the use of color Doppler helps to avoid vessels during the puncture. After puncture of the cavity and dilatation of the fistula tract, double-pigtail plastic stents or a lumen-apposing metal stent (LAMS) is placed across the lumen and extends into the necrotic cavity. 

LAMS, with its larger diameter compared to plastic stents, theoretically offers superior drainage and facilitates sequential direct endoscopic necrosectomy (DEN), potentially aiding in managing WON. The relative advantages of LAMS over plastic stents have not yet been determined with certainty. A recent meta-analysis found that the two methods had similar technical and clinical success rates [102]. Two recent RCTs and a comparative study using data from prospective trials comparing LAMS and double-pigtail plastic stents also failed to demonstrate any significant difference in technical or clinical efficacy, including the number of procedures needed [103,104,105]. In addition, the use of anchoring coaxial double-pigtail plastic stents within an LAMS has been shown to decrease the incidence of adverse events, including stent occlusion [106].

- Endoscopic necrosectomy

Endoscopists have increasingly attempted to perform DEN in patients with WON in addition to transmural drainage alone. DEN can be performed at the index procedure but is generally performed as subsequent procedures after the liquid component has been drained. To perform necrosectomy, a forward-viewing endoscope is used to access the necrotic cavity, which is then irrigated with saline, and loose necrotic debris is removed using a basket, snare, or other endoscopic accessories [107]. This procedure may be repeated until the cavity is cleared of debris.

A systematic review of 14 studies involving 455 patients with WON revealed that endoscopic drainage with necrosectomy achieved a clinical success rate of 81%, with an average of four endoscopic interventions per patient. The overall complication rate was estimated to be 36%, with a procedure-related mortality rate of 6% [108].

Hydrogen oxide lavage (median concentration, 3%; diluted with saline; volume, 20–1000 mL) has been introduced as an adjunctive therapy during endoscopic necrosectomy, with clinically acceptable results [109,110]. 

Percutaneous endoscopic necrosectomy is an alternative procedure performed through a percutaneous catheter drainage tract in patients with laterally positioned collections who have undergone percutaneous drainage. The catheter was upsized to 30 Fr, allowing access to the cavity by using a standard endoscope.

- Surgical debridement

Surgical pancreatic debridement can be performed using either minimally invasive or open techniques. Minimally invasive approaches are associated with less severe inflammatory response and lower physiologic stress than open surgery. The location of pancreatic necrosis, as determined via preoperative cross-sectional imaging, is a crucial factor in guiding the approach to debridement [90].

VARD and MIRP are the most commonly used techniques as the retroperitoneal approaches for draining retrogastric collections that extend to the left paracolic gutter. In addition, the VARD technique is a component of the minimally invasive surgical ‘step-up’ approach in the PANTER and TENSION trial [95,98]. These patients require preoperative percutaneous access to the retroperitoneal space. Long grasping forceps under direct vision of a videosope via an incision (5–7 cm) during VARD or two–three 30 Fr nephroscopes with forceps in the working channel without an incision during MIRP are used for debridement [111]. Debridement is typically repeated every 7–10 days until the necrotic cavity is free of debris and lined with healthy granulation tissue. A meta-analysis of VARD demonstrated a 64% success rate, 47% morbidity rate, and 14% mortality rate [112].

Surgical transgastric debridement, both laparoscopic and open, is suitable for patients with centrally located WON within the lesser sac. Compared to DEN, these surgical procedures are more definitive, but they also pose a higher risk of wound complications, such as pancreatic fistula [113,114].

The laparoscopic transperitoneal approach, which involves conventional intraperitoneal access, has several drawbacks, including the risk of peritoneal contamination and difficulty in reintervention due to scar tissue formation [115]. However, it remains a viable treatment option for patients with centrally located WON at the root of the mesentery, which is not amenable to transgastric or percutaneous techniques.

Open surgery is infrequently performed in patients with extensive necrosis inaccessible to both percutaneous and endoscopic drainage, in whom the step-up approach has failed, or in those with rare complications, such as bowel perforation, abdominal compartment syndrome, ischemic bowel infarction, or severe bleeding not amenable to angiographic coiling/embolization [116]. In a single-center study of 305 patients with necrotizing pancreatitis, 193 patients underwent endoscopic interventions, including endoscopic drainage alone or in combination with necrosectomy; of the patients who underwent early intervention within 4 weeks, 7% ultimately required open surgery due to refractory necrosis or complications such as bowel perforation [117].

- Comparison of endoscopic and minimally invasive surgical step-up approach

Both endoscopic and surgical ‘step-up’ approaches have been proven effective in managing infected necrosis. Subsequently, three RCTs compared the efficacy of the two interventions. The PENGUIN trial comparing endoscopic transluminal necrosectomy (*n* = 10) and various surgical necrosectomy techniques (*n* = 10) revealed a significant decrease in the inflammatory response (measured by interleukin-6) and the development of new-onset multi-organ failure in the endoscopic arm [113]. The TENSION trial compared endoscopic catheter drainage followed by endoscopic necrosectomy (if necessary) (*n* = 51) and percutaneous catheter drainage followed by VARD (if necessary) (*n* = 47) [98]. In the endoscopic and surgical groups, 43% and 51% of the patients required only catheter drainage, respectively. Furthermore, approximately one-third of patients in the endoscopic group underwent additional percutaneous catheter drainage or VARD. Conclusively, this study found no significant difference in the primary outcomes of a composite endpoint, including mortality and major morbidity at the 6 month follow-up (43% vs. 45%, *p* = 0.88); however, the endoscopic approach resulted in a shorter hospital stay (mean 53 vs. 69 days, *p* = 0.014) and significantly fewer pancreatic fistulae (5% vs. 32%, *p* = 0.001) [98]. The results of the long-term follow-up of the TENSION trial showed that the endoscopy group needed fewer reinterventions after the initial 6-month follow-up (7% vs. 24%, *p* = 0.038) [118]. The MISER trial compared minimally invasive surgery (laparoscopic or VARD) (*n* = 32) with the endoscopic step-up approach (*n* = 34). At six months, fewer patients in the endoscopic group had major complications or death (12% vs. 41%, *p* = 0.007) or fistulas (0% vs. 28%, *p* = 0.001) than those in the surgery group [114]. Unlike the TENSION trial, the MISER trial considered enteral and pancreatic fistula as major endpoints, explaining the primary difference in the conclusions between the two studies. In conclusion, while not superior in reducing death or major complications except pancreatic fistula, the endoscopic step-up approach seems to be the preferred treatment for infected necrotizing pancreatitis compared to the surgical approach.

## 4. Convalescent Treatment

### 4.1. Cholecystectomy in Gallstone AP

#### Timing of Cholecystectomy

Prophylactic cholecystectomy is commonly recommended during initial hospital admission for patients with acute biliary pancreatitis rather than after discharge [28]. This strategy aims to prevent recurrent episodes of pancreatitis, and same-admission cholecystectomy (within seven days) is proven to be more effective than interval cholecystectomy for the prevention of recurrent gallstone-related complications with cost-effectiveness in mild gallstone pancreatitis [119,120,121]. A recent study investigated the optimal timing and safety of cholecystectomy in patients with necrotizing biliary pancreatitis, with the aim of balancing recurrent biliary events with the risk of surgical complications. In conclusion, cholecystectomy in the absence of peripancreatic collections is thought to be preferably performed within 8 weeks after discharge [122]. 

### 4.2. Lipid-Lowering Medications for HTG-AP

Similar to other causes of AP, the initial management of HTG-AP involves fluid resuscitation, pain control, and nutritional support. After the acute episode, initiating diet and lifestyle changes along with hypolipidemic drugs is crucial to prevent further episodes of HTG-AP. Lipid-lowering medications such as fibrates, niacin, omega-3 fatty acids, and newer pharmacologic treatments like angiopoietin-like protein3(ANGPTL3) inhibitors, apolipoprotein C-III(ApoC-III) inhibitors, and pemafibrate can be used [123,124,125].

### 4.3. Alcohol Intervention in Alcoholic AP

Abstinence from alcohol can protect against recurrent AP [126]. In addition, the implementation of brief alcohol interventions during hospital admission, combined with repeated interventions, can improve the effectiveness of preventing AP recurrence [127]. However, there is still a lack of RCTs confirming the effectiveness of alcohol intervention only in patients with alcoholic AP. Further studies are needed to determine whether alcohol abstinence reduces recurrence and improves the prognosis of AP. 

## 5. Conclusions

Initial and convalescent treatments for AP are currently evolving based on recent clinical evidence. GDT with non-aggressive fluid resuscitation of buffered crystalloids has become the main strategy for AP treatment. Adequate pain control and early enteral feeding play important roles in the initial management of AP. Algorithm-based antibiotic use, rather than routine use for prophylaxis, makes it possible to tailor approaches to AP with clinical improvement. Except for the definitive role of urgent ERCP in acute biliary pancreatitis with cholangitis, a more conservative approach becomes widely valued. The treatment strategy for patients with necrotizing pancreatitis involves initial conservative management with a postponed drainage strategy in the early phase of the disease. In the late phase, a minimally invasive surgical or endoscopic “step-up” approach is typically employed. New pharmacological treatments, optimal timing of cholecystectomy in necrotizing biliary pancreatitis, and the efficacy of alcohol intervention need to be investigated in the future. 

## Figures and Tables

**Table 1:** Physiological parameters and their significance in GDT.

Parameters and Target	Significance in GDT
HR < 120/min	An elevated heart rate can indicate an imbalance between oxygen supply and demand, guiding therapeutic interventions in GDT. Persistent tachycardia might suggest inadequate resuscitation or ongoing inflammation.
MAP 65–90 mmHg	A consistent MAP is crucial for ensuring adequate blood flow to vital organs. In GDT, adjustments in fluid volume and vasopressor medications might be considered to maintain or achieve a target MAP, ensuring optimal organ perfusion.
CVP 8–12 cmH_2_O	It indicates the volume and filling status of the right atrium. In GDT, CVP is used to assess the patient’s volume status and right-sided cardiac preload, guiding fluid management.
UO ≥ 0.5 mL/kg/h	A decrease in UO is an early and sensitive indicator of reduced kidney perfusion. Maintaining adequate urine output is crucial in GDT as it provides valuable information on general tissue perfusion.
ScvO_2_ ≥ 70%	An indicator in assessing the adequacy of tissue oxygenation. A decrease in ScvO_2_ can suggest that tissue oxygen demand is exceeding supply. This could be due to decreased oxygen delivery (e.g., due to low cardiac output or hemoglobulin) or increased oxygen consumption (e.g., due to increased metabolic demand).
BUN < 25 mg/dL	An elevated BUN has been a useful prognostic biomarker of severe AP, reflecting acute renal injury in AP caused by a decrease in circulatory volume and direct injury mechanisms, which is facilitated by autodigestion and inflammatory cytokines [6,31]. However, a declining or normalized BUN level reflects recovery of renal perfusion and adequate resuscitation.
Hematocrit < 44%	Hemoconcentration (high hematocrit values) is linked with high fluid sequestration and increased viscosity, which might contribute to impaired pancreatic microcirculation. Therefore, hematocrit has long been identified as a marker associated with the development of pancreatic necrosis and persistent organ failure [32,33]. Fluid rate adjustment can be guided by the biochemical targets of hematocrit of 35–44% at 12 and 24 h after AP onset.
Lactate	Lactate level increases when aerobic cellular respiration is impaired with a switch to anaerobic metabolism. Elevated lactate level has been considered a well-recognized biomarker of tissue hypoxia/hypoperfusion in critically ill patients.

**Table 2:** Summary of recent RCTs comparing the protocol of fluid resuscitation in AP.

Reference	Participants (N)	Aggressive Resuscitation	Non-Aggressive Resuscitation	Effect of Aggressive Resuscitation
Mato et al., 2009 [44]	Severe AP lesser than 72 h onset (76)	10–15 mL/kg/h	5–10 mL/kg/h	Harmful, more sepsis, mortality, mechanical ventilation, and ACS.
Mato et al., 2010 [45]	Severe AP lesser than 24 h onset (115)	Rapid hemodilution with goal hematocrit < 35% at 48 h	Slow hemodilution with goal hematocrit > 35% at 48 h	Harmful, more sepsis, and mortality
Wu et al., 2011 [46]	Any severity AP (40)	GDT with 20 mL/kg bolus → 1.5 or 3 mL/kg/h of LR or NS	LR or NS adjusted by physician	Similar in SIRS and CRP at 24 h
Buxbaum et al., 2017 [24]	Predicted mild AP (60)	20 mL/kg bolus over 2 h → 3 mL/kg/h infusion of LR	10 mL/kg bolus over 2 h → 1.5 mL/kg/h infusion of LR	Beneficial, more clinical improvement, and less persistent SIRS and hemoconcentration
		At timepoint (12, 24, 36 h) If hematocrit, BUN, or creatinine increased, 20 mL/kg bolus → 3 mL/kg/h infusion If labs were decreased and pain relived, 1.5 mL/kg/h infusion and start diet		
Cuéllar-Monterrubio et al., 2020 [47]	Any severity AP, more than 24 h onset (88)	20 mL/kg bolus → 3 mL/kg/h (first 24 h) → 30 mL/h (next 24 h) of HS	20 mL/kg bolus (only if hypovolemia) → 1.5 mL/kg/h (first 24 h) → 30 mL/h (next 24 h) of HS	No benefit, no difference in persistent SIRS, pancreatic necrosis, respiratory complications, AKI and LOS
De-Madaria et al., 2022 [23]	Mild AP, lesser than 24 h onset (249)	20 mL/kg bolus → 3 mL/kg/h infusion of LR	10 mL/kg bolus (only if hypovolemia) → 1.5 mL/kg/h infusion of LR	Harmful, more fluid overload
		At timepoint (3,12, 24, 48, 72 h) If hypovolemia → 20 mL/kg bolus → 3 mL/kg/h If normovolemia → 1.5 mL/kg/h If fluid overload → decrease or stop	At timepoint (3,12, 24, 48, 72 h) If hypovolemia → 10 mL/kg bolus → 1.5 mL/kg/h If normovolemia → 1.5 mL/kg/h If fluid overload → decrease or stop	

AP, acute pancreatitis; ACS, abdominal compartment syndrome; LR, lactated Ringer’s; NS, normal saline; SIRS, systemic inflammatory response syndrome; CRP, C-reactive protein; BUN, blood urea nitrogen; HS, Hartmann’s solution; AKI, acute kidney injury; LOS, length of stay.

## Data Availability

Data sharing is no applicable.
